# Editorial: Maternal and infant nutrition: impact on breast milk, infant gut microbiota and health development, volume II

**DOI:** 10.3389/fnut.2026.2000843

**Published:** 2026-10-29

**Authors:** Valeria Calcaterra, Xinran Liu, Yalin Zhou, Gianvincenzo Zuccotti

**Affiliations:** 1 Pediatric and Adolescent Unit, Department of Internal Medicine, University of Pavia, Pavia, Italy; 2 Pediatric Department, Buzzi Children's Hospital, Milan, Italy; 3 Department of Clinical Nutrition, Peking University People's Hospital, Beijing, China; 4 Department of Nutrition and Food Hygiene, School of Public Health, Peking University, Beijing, China; 5 Department of Biomedical and Clinical Science, University of Milan, Milan, Italy

**Keywords:** breast milk, gut microbiota, health, infant, maternal, nutrition

## 1 Introduction

Nutrition during pregnancy, lactation, and early infancy represents a major interface between maternal health, infant development, and lifelong disease susceptibility. The first 1,000 days are characterized by exceptional biological plasticity, during which nutritional, metabolic, microbial, environmental, and psychosocial exposures may influence growth, immune maturation, neurodevelopment, and cardiometabolic trajectories. Within this framework, human milk should be regarded not simply as a source of nutrients, but as a dynamic biological system containing lipids, proteins, carbohydrates, micronutrients, hormones, immune mediators, microorganisms, and other bioactive components that interact with the developing infant.

The Research Topic “*Maternal and Infant Nutrition: Impact on Breast Milk, Infant Gut Microbiota and Health Development, Volume II*” was conceived to extend this integrated perspective. The contributions collectively move beyond a nutrient-centered view of early-life nutrition and highlight the mother–infant dyad as an interconnected biological and social system. Maternal diet and health, human milk composition, microbial colonization, infant growth, feeding behavior, and healthcare and sociocultural environments emerge as interacting determinants rather than independent exposures.

## 2 Maternal determinants and the biological complexity of human milk

Two complementary studies by Alodhaybi et al. (a). examined distinct maternal determinants of human milk composition. The first investigated maternal hypothyroidism in relation to human milk macronutrients and fatty acid composition, illustrating how maternal endocrine status may influence the nutritional environment experienced by the breastfed infant. In a separate cohort study, Alodhaybi et al. (b) assessed maternal dietary intake and showed that, while milk macronutrient composition appears relatively preserved, fatty acid profiles may be more responsive to maternal diet. Considered together, these two studies emphasize that human milk combines biological stability with selective plasticity.

This concept is further expanded by Selvalatchmanan et al., who investigated the human milk lipidome in a multi-ethnic Asian cohort and identified associations with both maternal ethnicity and antenatal anxiety. Their findings broaden the determinants of milk composition beyond conventional nutritional exposures, suggesting that biological, psychosocial, and population-related factors may converge on milk molecular composition. Such data support the emerging view of human milk as a potential biological interface through which maternal phenotype and environment may influence early development.

Human milk is equally dynamic from a microbial perspective. Xie et al., studying milk samples across different stages of lactation, showed marked temporal changes in microbial community structure. Lactation stage was the strongest measured determinant of microbiome variation, while delivery mode and intrapartum antibiotic exposure exerted more prominent effects during early lactation. These findings caution against viewing the human milk microbiome as a fixed entity and underscore the importance of considering timing when studying maternal–infant microbial transmission.

Extending this perspective from milk to the infant gut, Liu et al. longitudinally followed 67 mother–infant dyads during the first year of life, documenting substantial age-related microbiota maturation associated with feeding mode, delivery mode, complementary feeding timing, breast-milk density, and maternal dietary quality. These findings support the interconnected role of maternal nutrition and infant feeding in early microbial development.

Together, these studies indicate that human milk composition and early microbial development cannot be fully understood through isolated nutritional or microbial measures. Integrating lipidomics, microbiome profiling, maternal endocrine and dietary characteristics, and psychosocial factors may provide a more comprehensive view of the biology of breastfeeding.

## 3 Early-life programming: growth, micronutrients, microbiota, and cardiometabolic health

The contributions also reinforce the importance of developmental timing and individual susceptibility. Zhang et al. reported nonlinear and sex-specific associations between maternal vitamin D concentrations during pregnancy and childhood growth trajectories extending to 6 years of age. Rather than supporting a simple linear exposure–outcome relationship, these results point toward thresholds, developmental windows, and sex-dependent responses that deserve consideration in future nutritional studies.

A similar developmental perspective emerges from Ge et al., who examined iron deficiency at 6 months of age. Their findings highlight rapid postnatal weight gain as an important pathway linking male sex with increased iron-deficiency risk. This observation illustrates a broader principle: optimal growth cannot be evaluated independently of micronutrient requirements. Accelerated growth may increase nutritional demands and therefore modify vulnerability to deficiency even in apparently healthy infants.

At the mechanistic level, Mei et al. used an experimental model of maternal high-fat diet to examine the effects of perinatal phlorizin exposure on offspring metabolism and gut microbiota. Their findings provide biological support for the concept that maternal metabolic exposures can influence offspring phenotype through pathways involving microbial ecology and metabolic regulation. Although translation from animal models requires caution, such studies are important for identifying candidate mechanisms linking maternal nutrition to later cardiometabolic risk.

This mechanistic dimension is integrated by Rojas-Rodríguez and Jaramillo-Romero, who review evidence linking the gut microbiota during the first 2 years of life with the early programming of obesity, type 2 diabetes, and hypertension. The microbiota may act as an interface between feeding, environmental exposures, immune development, and host metabolism rather than as an isolated determinant. Early dysbiosis should therefore be understood within a broader developmental network encompassing diet, growth, inflammation, microbial metabolites, and metabolic signaling.

The implication is important: prevention of non-communicable disease may begin much earlier than the appearance of conventional cardiometabolic risk factors. However, microbial signatures should not yet be interpreted as deterministic biomarkers. Longitudinal studies integrating microbiome, metabolomics, dietary assessment, growth trajectories, and clinical outcomes are required before microbiota-directed interventions can be incorporated into routine preventive strategies.

## 4 From biological evidence to breastfeeding support and public health

A major strength of this Research Topic is that it extends beyond biological mechanisms to the environments in which maternal and infant nutrition occur. Shahzad et al., through the CHAMP prospective cohort in a malnutrition-endemic region of Pakistan, describe the complex coexistence of maternal dietary inadequacy, neonatal nutritional vulnerability, feeding practices, and healthcare-related factors. Their work reinforces that maternal and infant nutrition cannot be separated from food availability, socioeconomic conditions, healthcare access, education, and local cultural practices.

The importance of perception and social context is further illustrated by Haq et al., whose qualitative investigation of perceived insufficient breast milk highlights how maternal beliefs, family influences, uncertainty regarding milk adequacy, and limited professional support may affect breastfeeding continuation. Biological capacity alone does not determine breastfeeding success; maternal confidence and the surrounding support system are critical components of lactation care.

Rojas-Rodriguez et al. examined early breastfeeding initiation in Southern Ecuador, highlighting associations with maternal and perinatal conditions and emphasizing the importance of the immediate postnatal period and maternity-care practices in supporting the timely establishment of breastfeeding.

Balakrishnan et al. similarly move beyond binary indicators such as “breastfed” vs. “not breastfed” by identifying distinct infant feeding profiles during the first 6 months postpartum. Their approach illustrates how longitudinal feeding trajectories may better capture real-world behavior and may provide a more informative framework for evaluating breastfeeding promotion programs.

Complementary feeding represents a critical transition increasingly shaped by household and structural factors. Vakilna et al., studying children aged 6–23 months in Gujarat, identified gaps in dietary diversity and age-appropriate feeding, together with maternal, household, and practical barriers, highlighting the need for sustained caregiver and community support.

At the healthcare-system level, Liu and Liu challenge the traditional “light-diet” approach to postpartum hospital nutrition in China, emphasizing that culturally established practices do not necessarily guarantee adequate nutrient density. Their Perspective illustrates the need to translate nutritional science into measurable standards for postpartum care and to recognize maternal nutrition as an integral component of infant health promotion.

Finally, Biagioli et al. propose a One Health Decalogue for supporting infant microbiota when exclusive breastfeeding is not possible. Importantly, such an approach does not diminish breastfeeding as the biological reference for infant nutrition. Rather, it acknowledges clinical reality and expands preventive care toward formula composition, complementary feeding, environmental exposures, antibiotic stewardship, lifestyle, and family support. This broader perspective is consistent with the overall message emerging from the Topic: early-life health is generated by interacting biological, environmental, and social determinants.

The translational dimension of infant feeding is further illustrated by Al-Fakhroo et al., who explored perceptions of human milk oligosaccharides and HMO-enriched formula among parents and healthcare professionals in Qatar. Limited knowledge and cautious acceptance highlighted the need for evidence-based communication and professional education to support informed feeding decisions.

## 5 Emerging perspectives

Taken together, the contributions to this Research Topic highlight the close interconnection between maternal health, human milk composition, early-life programming, and the broader context in which infant feeding occurs. Maternal endocrine and metabolic status, dietary quality, psychosocial factors, and sociocultural conditions may all shape the nutritional environment experienced by the infant.

Human milk is increasingly recognized as a dynamic biological system rather than a uniform nutritional substrate. Its lipid, fatty acid, microbial, and bioactive composition varies with maternal characteristics and stage of lactation, underscoring the value of longitudinal approaches that capture temporal changes.

Early-life programming is similarly multidimensional. Sex, growth velocity, micronutrient status, microbiota development, breastfeeding duration, milk composition, maternal diet, and complementary feeding may interact across critical developmental windows. Future precision approaches will therefore require integration of biological, nutritional, and clinical information, together with the social and behavioral context in which feeding practices evolve.

Translating this knowledge into improved health outcomes will also depend on supportive family, healthcare, and public-health environments. Appropriate infant nutrition reflects not only maternal choices, but also professional guidance, hospital practices, socioeconomic conditions, cultural factors, and access to support. Effective interventions should therefore consider the mother–infant dyad within this broader biological and social framework.

## 6 Conclusions

Volume II supports a systems-based view of maternal and infant nutrition, in which maternal health, human milk, feeding practices, and microbial and metabolic development are considered as interacting components of early-life health within specific social, cultural, and healthcare contexts.

The next challenge is to connect these dimensions through large longitudinal cohorts integrating repeated dietary and human milk assessments, multi-omics and microbiome profiling, objective growth and metabolic phenotyping, and detailed characterization of environmental and social determinants. Particular attention should be given to critical developmental windows, sex-specific responses, and populations facing nutritional or socioeconomic disadvantage.

Ultimately, improving lifelong health requires an integrated strategy that begins before birth, supports maternal health throughout pregnancy and lactation, protects and promotes breastfeeding whenever possible, provides evidence-based alternatives when breastfeeding is not feasible, and recognizes early nutrition as a shared responsibility of families, healthcare systems, and society.

